# A Comparative Investigation of the Reliability of Biodegradable Components Produced through Additive Manufacturing Technology

**DOI:** 10.3390/polym16050615

**Published:** 2024-02-23

**Authors:** Amged ElHassan, Waleed Ahmed, Essam Zaneldin

**Affiliations:** 1 Mechanical and Aerospace Engineering Department, College of Engineering, UAE University, Al Ain P.O. Box 15551, United Arab Emirates; 201450104@uaeu.ac.ae; 2 Engineering Requirements Unit, College of Engineering, UAE University, Al Ain P.O. Box 15551, United Arab Emirates; 3 Civil and Environmental Engineering Department, College of Engineering, UAE University, Al Ain P.O. Box 15551, United Arab Emirates

**Keywords:** failure, 3D Printing, defects, reliability, biodegradable

## Abstract

Using the linear elastic finite element method, we investigated how defects significantly influence the integrity of 3D-printed parts made from biodegradable material by experimental techniques and numerical simulations. A defective flaw was incorporated into the tensile test dog-bone sample using Computer-Aided Design and processed by slicing software. Three distinct raster angles examine two sets of samples, one featuring intact specimens and the other with the introduced defects. An open-source 3D printer was used to fabricate both sets of samples, utilizing biodegradable PLA material. In finite element analysis, we employed a highly detailed model that precisely accounted for the geometry and dimensions of the extruded 3D-printed filament, accurately replicating the actual configuration of the 3D-printed samples to an extent. Our study involved a thorough comparative analysis between the experimental results and the FEA simulations. Our findings uncovered a consistent trend for the intact and defective samples under tensile load. Specifically, in the intact case, the samples with a zero-degree raster orientation presented the highest resistance to failure and displayed minimal elongation. Remarkably, these conclusions paralleled our observations of the defective samples as well. Finite element analysis revealed that the stresses, including Principal, Max shear, and Von Mises, were remarkably higher at the 3D-printed samples’ outer surface than the inner layers, reflecting that the failure starts at the outer surface since they exceeded the theoretical values, indicating a significant discrepancy between the simulated and anticipated values.

## 1. Introduction

**Figure 1:**
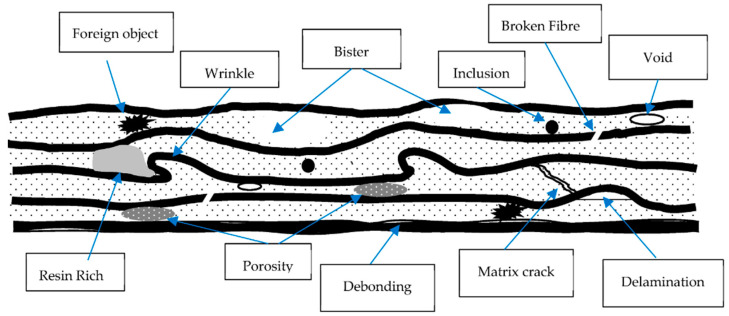
Common defects in the material [8].

3D printing, alternatively referred to as additive manufacturing, has rapidly emerged as a game-changing and revolutionary technology within manufacturing and engineering [1]. In stark contrast to conventional subtractive manufacturing methods, which necessitate material removal from a solid block, additive manufacturing constructs objects layer by layer. This innovative approach grants unparalleled design flexibility and customization, ushering in a new era of creative possibilities [2]. This pioneering technology has garnered extensive adoption across various industries, from aerospace and healthcare to automotive and consumer goods. Its ability to fabricate intricate geometries, expedite prototyping, and offer cost-effective customization has ushered in profound product development and manufacturing process transformation [3]. Despite its manifold advantages, additive manufacturing encounters challenges related to material selection, post-processing requirements, and quality control issues [4]. Nevertheless, ongoing research and development endeavors persistently push the boundaries of this technology, hinting at a future where additive manufacturing will be pivotal in reshaping how we conceive, craft, and manufacture products across a broad spectrum of industries [5]. During the FDM 3D printing process, the raster angle is the angle at which each successive layer of material is placed [6]. It is sometimes referred to as layer orientation or print orientation. This angle can be adjusted to improve the look of the printed product. The concept of raster angle significantly impacts printed items’ quality, strength, and overall performance in 3D printing technology. The direction of the consecutive layers or routes of material deposition during the additive manufacturing process is referred to as the raster angle [7]. Figure 1 represents the general defects in the material.

While it may appear an essential characteristic, it has substantial consequences for the final output. The raster angle can significantly impact the printed object structural integrity, surface polish, and even anisotropic qualities [9]. An optimized raster angle is critical for obtaining required mechanical properties, lowering the probability of defects, and improving the overall performance of 3D-printed items [10]. Material strength, surface finishing, printing time, and support material, among other factors, all influence the raster angle [11]. The raster angle describes the angle at which subsequent layers are deposited or sintered in relation to the base or build platform. This angle has a considerable impact on the mechanical qualities, surface finish, and overall quality of the printed object [10,12]. The selection of raster angle in the context of additive manufacturing and 3D printing is inextricably linked to the mechanical material qualities of the chosen material [13]. Furthermore, the raster angle might influence fatigue resistance, with some orientations potentially increasing vulnerability to fatigue failure due to directional flaws [8,14]. Thus, careful consideration of raster angle is critical in optimizing the mechanical performance of additively built components, balancing strength, anisotropy, and fatigue resistance in accordance with specific application requirements [15].

The mechanical behavior of a material, which includes parameters such as tensile strength, elasticity, and thermal conductivity, substantially impacts how it responds to 3D printing [16]. The raster angle, which determines the orientation of subsequent layers or courses of material deposition, is critical in adequately harnessing various material features [13]. For example, aligning the raster angle with the material intrinsic strength can improve the printed product overall structural integrity [17]. On the other hand, misaligned raster angles may cause weaknesses or anisotropic behavior, in which the mechanical properties of the printed item change along different axes. As a result, intelligent raster angle selection, informed by a thorough grasp of the material properties, is critical for optimizing mechanical performance and guaranteeing that 3D-printed components match the appropriate requirements and standards [17,18]. Furthermore, such misalignment frequently exacerbates the anisotropic character of additive manufacturing, as mechanical qualities change greatly depending on the orientation of the raster angles in relation to the applied force [19,20]. As a result, items with mismatched raster angles may have uneven mechanical performance across multiple loading directions, rendering them unsuitable for applications that need uniform strength and reliability [21]. Thus, meticulous raster angle alignment is required to achieve excellent mechanical characteristics and overall part quality in additive manufacturing methods [22].

Many studies have been conducted to investigate the mechanical behavior of FDM-printed products. Xinzhou Zhang et al. [23] investigated a fused deposition modeling (FDM) technique to examine the mechanical characteristics of Polylactic acid (PLA) and aluminum with fiber-reinforced PLA composite material samples. In addition, the impact of raster angle on the thermal, mechanical, and tensile characteristics of FDM printed and AI/ PLA was also observed. Q. Sun et al. [24] investigated the mechanism controlling the bond formation among extruded polymer filaments in the fused deposition modeling process. They also examined that the thermal bonding determined the integrity and mechanical qualities of the resulting prototypes. A.C. Abbott et al. [25] conducted tensile tests on ABS coupons printed in two orientations in order to determine bond strength between and within layers as a function of print settings. The print parameters studied were extruder temperature, print speed, and layer height. An infrared camera was utilized to monitor the heat history of interlayer bond lines during the printing process. Bandar Aloyaydi et al. [26] examined the impact of infill patterns on mechanical response of 3D-printed specimens by using LVI and compression tests. They found that the triangular pattern produced the highest absorbed energy in the LVI test (penetration energy 7.5 J, stiffness 668.82 N/mm) due to more sheared/contact layers perpendicular to the impactor (hemispherical insert), while the grid pattern had the highest compressive strength (72 MPa) due to more layers aligned along the compression loading direction. The SEM fracture surface picture of Triangular IP shows good raster and layer bonding, reduced voids, increased circular beach marks, and no ratchet lines, resulting in improved mechanical properties. Chamil Abeykoon et al. [27] mainly focused on examination of the properties of 3D-printed specimens (mechanical, thermal, and morphological) under diverse processing settings, including infill pattern, density, and speed, as well as printing materials. Xia Gao et al. [28] examined the influence of molecular diffusion, entanglements, and crystallization behaviors in FFF-printed parts on semi-crystalline polymer interlayer bond quality. In addition, Polylactide (PLA) printing materials are modified with different molecular weights of polyethylene glycol (PEG) to improve interlayer bond quality and reduce mechanical anisotropy. Piotr Wolszczak et al. [29] investigated the issue of heat distribution in FDM 3D printing. They examined that the temperature distribution of a material impacts shrinkage and crystallization, affecting its dimensional correctness and strength. EvaBozó et al. [30] investigated the use of bioplastic such as PLA, polyhydroxybutyrate (PHB) blends, pyrolyzed lignin (PL), and multi walled carbon nanotubes (MWCNTs) in electronics component fabrication processes like plasma treatment, dip coating, inkjet and screen printing, hot mixing, extrusion, and molding. Jomin Thomas et al. [31] reviewed on approach to polymer-based sustainability concerns rely on technical knowledge and facts. Four key subjects of relevance were chosen and reviewed without bias. The basic comprehension of sustainability-related vocabulary is assessed at the start to ensure fundamental awareness. The rise of bio-based polymer products was analyzed against their unique biodegradability challenges. Syed Fouzan Iftekar et al. [32] provided a critical review of the recent advancement in 3D printing technology, with an emphasis on materials and applications in the industrial business. They also emphasized the importance of further developing 3D printing technology in order to overcome its current constraints. 

M.A. Tan et al. [33] deliberated the mechanical properties of PLA, utilizing 3D printers to investigate the relationship between infill density and raster angle. More complicated 3D design objects were created via 3D printing. To analyze the properties of pure PLA, the tensile test was used. The greatest tensile strength and Young modulus for pure PLA were 28.926 MPa and 1262.7 MPa, respectively, for 0° raster angles with 100% infill density. Betül Gülçimen Çakan [34] explored the parameters that influence tensile and surface roughness qualities. They discovered that as the raster angle increased, the ultimate tensile strength (UTS) dropped; thus, the 0° raster angle produced the highest strength where the tensile applied load direction was parallel to the raster. Wenzheng Wu et al. [17] investigated the impact of the thickness of the layer and raster angle on the mechanical characteristics and qualities of 3D-printed products.

They also examined tensile strength, compressive strength, and bending strength with three various material specimens with three different thicknesses and raster angles using a polyether-ether-ketone (PEEK) 3D printing approach. They observed the best mechanical properties at 300 mm layer thickness and 0-degree raster angle. P. Biswas et al. [35] developed a computational model based on the microstructure of 3D-printed ANS polymers to examine the orthotropic elastic characteristics utilizing micromechanics of a representative volume element (RVE) approach. Sunil Bhandari et al. [36] developed a homogenized and linear elastic continuum FEA model in which the simulation was carried out to homogenize the cellular structure. Physical experiments were also conducted to validate the accuracy of the outcomes generated from the continuum FEA model.

Mohammed Algarni [37] evaluated the impact of raster angle and moister content percentage on the mechanical characteristics of 3D-printed PLA material samples. They also investigated the three different raster angles and found that the PLA material was varied to see how it impacted mechanical characteristics. Hamza Qayyum et al. [18] proposed material test specimens with different raster angles and infill patterns to examine the influence of raster angle on the plane and edgewise flexure strength of ABS material. They observed that the 0-degree raster angle printing delivered the best in-place and edgewise flexure strength. Ruoxiang Gao et al. [38] investigated the impact of raster angle and infill density which are more effective parameters, using a single factor experiment. They examined from the obtained outcomes that the raster angle 0/90 degree and density of 50% has the overall best consequences in terms of warpage deformation, tensile strength, and specific strength according to obtain an evaluation. Hassan Gonabadi et al. [10] proposed a numerical homogenization approach to quantify the impact of printing process parameters on the elastic response of 3D-printed products with cellular lattice structures.

The present study explored the impact of manufacturing processes, specifically focusing on the angles at which layers are deposited in 3D printing, with a primary emphasis on Fused Deposition Modeling (FDM) technology. The objective was to explore how these factors influence the printed components’ mechanical properties and overall structural integrity. Furthermore, in this research, Linear Elastic Finite Element Analysis (FEA) was utilized to assess the failure behaviors exhibited by the printed components. This revealed a substantial and essential finding, offering insights into the fundamental factors responsible for failures in 3D-printed parts resulting from various raster angle production processes supported by FEA.

## 2. Material Characteristics

The 3D-printed samples were produced utilizing the Ultimaker S5 3D printer to explore the influence of raster angles on the mechanical properties of defective 3D-printed parts, with a primary focus on a through-thickness defect. These specimens were produced using standard PLA filament material; details regarding the material properties of the filament employed in this study can be found in Table 1.

Moreover, this study examined various 3D-printed specimens produced at varying raster angles to assess their mechanical behavior and failure mechanisms. Tensile testing was employed as the method of choice to evaluate material properties under tensile loading conditions. Such tests yield critical data on tensile strength and stiffness, predominant in scenarios where materials undergo diverse loads or when ensuring structural integrity is most important.

**Figure 2:**
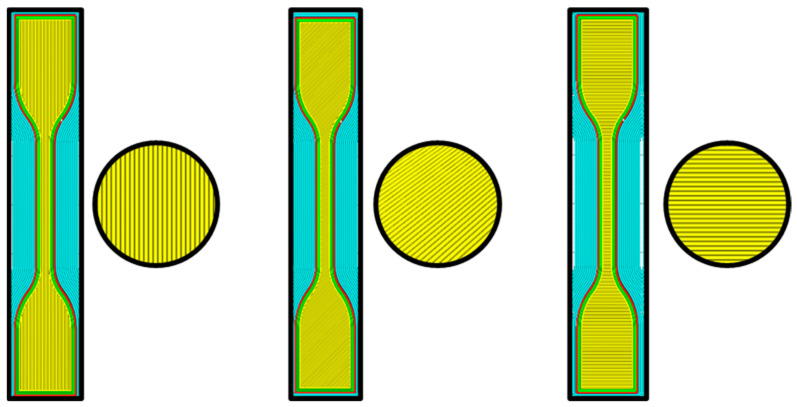
Intact samples at 0, 45, and 90 degrees (from left to right).

This strategy comprehensively evaluated how various manufacturing and loading conditions impact the structural characteristics and failure mechanisms. We adhered to the ASTM D638 standard [40] to configure our tensile test specimens, both intact and containing flaws, across different raster angles: 0 degrees, 45 degrees, and 90 degrees. Figure 2 visually illustrates these sample configurations and their respective dimensions. This method enabled us to acquire valuable understanding regarding the comprehensive structural performance of the scrutinized components.

**Figure 3:**
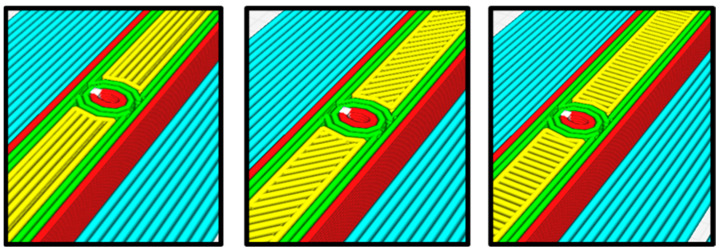
A 3D-printed flawed sample at different raster angles (0, 45, and 90 degrees from left to right).

Compliance with established testing standards was essential to maintain methodological consistency and standardization, which, in turn, facilitated precise comparisons of tensile properties between the intact and flawed specimens. Particularly, it was observed that the actual 3D-printed elliptical flaw exhibited dimensions somewhat smaller than those anticipated initially as shown in Figure 3. This discrepancy arose from the limitations of the 3D printer and the certain properties of the extrusion nozzle employed, including factors such as layer height, nozzle size, and extrusion width. It is important to note that FDM parts may undergo some degree of shrinkage during the cooling process, potentially contributing to the observed reduction in flaw size compared to the initial design.

The tensile test specimens were purposely manufactured with a definite through-thickness flaw arrangement. This chosen configuration for the flaw comprised a length of 1 mm, and the flaw was assumed on an elliptical form. Specifically, the elliptical defect exhibited a one-to-half ratio between its major and minor axes, signifying that its width was half the length, thus creating an elongated elliptical shape. By employing this particular flaw geometry, we analyzed how the specimens would respond mechanically when subjected to tensile forces. This specific configuration enables us to concentrate on understanding how the flaw affects the tensile properties of the samples, offering significant insights into how 3D-printed components perform when experiencing tensile loads. Our observations revealed distinguished differences in the failure behavior between the flawed and defect-free specimens, particularly concerning the failure location, which we will elaborate. In the case of the flawed specimens, the failure was exhibited precisely at the midsection of the dog-bone-shaped piece, right at the site of the flaw. This observation suggests that the flaw functioned as a stress concentrator, inducing a localized elevation in stress levels. Consequently, the material reached its breaking point at that specific location, with the flaw propagating and eventually resulting in the overall failure of the specimen. Concerning the undamaged, flawless specimens, the absence of stress concentration at the flaw allowed for a different failure pattern. Instead, the failure location shifted towards the shoulder radius of the sample. This region typically encounters elevated stress concentrations due to the change in section dimensions. Stress concentration arises when there is a sudden alteration in geometry, such as a reduction in cross-sectional area or a sharp change in curvature. In this scenario, the shoulder radius exhibited a higher stress level, ultimately surpassing the material strength and leading to failure in that specific area. This study emphasized the printing parameters to ensure the consistent and dependable fabrication of all specimens, thus maintaining the quality of the printed samples. Table 2 comprehensively summarizes the standard printing parameters utilized for the specimens with flaws and those without. These parameters have been widely adopted in the commercial production of polymeric 3D-printed components, having undergone extensive optimization and scrutiny by numerous researchers. As a result, no modifications were made to the manufacturer preset parameters, ensuring a reliable reference setting. We thoroughly followed these parameters to minimize printing process deviations and ensure uniform sample quality under all experimental conditions. These parameters incorporate a range of factors, including layer thickness, infill density, print speed, and temperature, all of which combine to uphold the overall strength and dependability of the printed specimens.

In order to delve into the impact of varying raster angles in 3D-printed specimens on failure behavior and overall performance, a systematic analysis was carried out. This involved a thorough investigation comprising three distinct sets of 3D-printed samples. An essential aspect of this inquiry was the meticulous control and manipulation of the flaw initiation process, allowing for a detailed assessment of its influence on the structural integrity of the samples. In the 3D printing process, we employed Simplify 3D (v5), a slicing software, to generate three distinct raster configurations at 0, 45, and 90-degree angles. Simplify 3D functions by taking the designed CAD model and slicing it into multiple layers, then stacked on top of each other. Subsequently, it creates a G-code file that instructs the 3D printer during the physical part fabrication. The utilization of Simplify 3D in this research ensured a standardized and dependable approach to slice the CAD models. This, in turn, improved the reproducibility and precision of the specimens under examination, contributing to the consistency and accuracy of our findings. Producing small holes in Fused Deposition Modeling (FDM) 3D printing can pose significant difficulties and yield unsatisfactory results. The constraints inherent to FDM technology, including nozzle diameter and layer height limitations, render creating intricate features, such as tiny holes and complex design flaws, a challenging endeavor [13]. Furthermore, even when employing a smaller nozzle size, the resolution remains constrained due to factors like filament flow rate, material characteristics, and the mechanical capabilities of the printer.

Furthermore, the layer height also influences the precision of small features [8]. Decreasing the layer height enhances vertical resolution but can substantially extend the printing duration, while excessively minimal layer heights can lead to inadequate layer adhesion. Additionally, the material properties play a crucial role in the capability to create small holes. Certain materials commonly used in FDM 3D printing, such as thermoplastics, may have constraints related to their flow and viscosity, posing challenges when aiming for exceptional detail [14]. Thus, this investigation was restrained to working within the dimensions of elliptical through-thickness flaws measuring 1 mm by 0.5 mm. These particular dimensions were the only ones possible through printing, while ratios of 1 to 0.3 and 1 to 0.1 were excluded from the experimental scope due to the limitations posed by the Fused Deposition Modeling (FDM) 3D printer utilized. Although attempts were made to fabricate flaw geometries with these ratios as designed, these efforts proved unsuccessful.

In this specific study, the 3D-printed specimens were produced with a solid composition, utilizing a linear infill pattern set at 100% density. The decision to maintain uniformity in infill pattern and density was intentional, as the study primary goal was not to explore the effects of different infill patterns or densities on failure performance. Instead, the primary focus was directed toward evaluating the significance of differences between specimens before and after flaws were introduced, as well as assessing the potential consequences on the overall structural integrity of the specimens stemming from the adoption of varying raster angles. The samples experienced slow-speed testing at a rate of 1 mm/min using a uniaxial tensile testing rig. To distinguish the samples according to their manufacturing processes, specific identification methods were implemented. The samples were then classified into the following categories:

- The 3D-printed undamaged specimens: These specimens were created without any defects and played the role of a reference point for comparison with the other types of samples.
- The 3D-printed flawed specimens: In this group, all specimens were intentionally introduced with a centrally located through-thickness flaw, possessing an aspect ratio of one to half, employing 3D printing technology. This precise flaw geometry was consistently incorporated into each of these specimens.

In examining both intact and flawed specimens, we took into account three varying raster angles as part of our analysis, with the aim of understanding how these angles affect the occurrence of failure. In a broader context, the quality of 3D-printed components can be influenced by numerous factors. Ouassil and colleagues [22] investigated how different variables impact the mechanical characteristics of 3D-printed polyetherimide parts along the Z-direction. Their research underscored that the Fused Filament Fabrication process has limitations when it comes to attaining optimal mechanical properties in this particular orientation. The analysis revealed that the most pivotal parameter for optimization is the adjustment of printing speed. Additionally, the study observed that the specimen condition, whether annealed or in its printed state, had minimal impact, primarily due to the amorphous nature. Furthermore, another investigation [15] investigated the influence of process variables and strain rate sensitivity on the mechanical properties of Onyx, a composite material comprising nylon and carbon fiber manufactured using Fused Filament Fabrication (FDM). The research findings demonstrated that adjustments in printing parameters, including adjustments in platform temperature and print speed, substantially impacted the tensile properties of the 3D-printed samples. Elevating platform temperatures resulted in increased Young’s modulus and tensile strength but a reduction in failure strain. Conversely, higher print speeds improved tensile strength but led to a decrease in failure strain. Moreover, the strain rate played a significant role in determining the failure mechanism, shifting from ductile to brittle behavior as the elongation rate increased.

### Investigation Hypothesis

In this comprehensive analysis, we have systematically addressed hypotheses covering the states of 3D printing processes, finite element analysis (FEA), and experimental work. Each set of hypotheses has been discussed to study specific aspects within their respective domains, providing a universal exploration of material behaviors, structural responses, and the real-world implications of our findings.

FDM 3D Printing Hypotheses:

1. Consistent Layer Adhesion Properties: Varied printing parameters do not compromise the consistent layer adhesion properties, ensuring robust structural integrity.
2. Fixed Printing Temperature and Print Speed: Maintaining a fixed printing temperature and speed contributes to standardized material deposition, enhancing print quality and structural uniformity.
3. Uniform Infill Density Over the Samples: Ensuring uniform infill density across printed samples results in consistent mechanical properties, mitigating structural variations within the printed objects.
4. Uniform Filament Material Properties: Homogeneous filament material properties contribute to predictable and reproducible mechanical behavior across the printed samples.
5. Printed Samples are Defect-Free: Precise quality control measures during the 3D printing process eliminate defects, ensuring the reliability and accuracy of printed components.
6. Dimensional Stability: Controlled printing conditions lead to dimensional stability, minimizing distortions and deviations in the final printed objects.

FEA Hypotheses:

7. Linear Elastic Analysis: The linear elastic analysis assumption represents the deformation behavior of FDM 3D-printed structures under applied loads.
8. Isotropic Properties: Treating the material as isotropic simplifies the analysis without compromising the accuracy of predicting structural responses.
9. Layers Overlapped at 0.01 mm: Overlapping layers at a specific distance ensures a realistic representation of the FDM printing process and its impact on structural integrity.
10. Adhesion Characteristic is Neglected: Neglecting adhesion characteristics in FEA does not significantly affect the accuracy of predicting stress and strain distributions in FDM printed components.
11. Analysis Performed at the Max Experimental Load: Conducting FEA at the maximum experimental load provides insights into the structural performance of printed objects under extreme conditions.
12. Consistent Mesh Density Over Samples: Maintaining a consistent mesh density across samples ensures a reliable and comparable analysis of structural behavior.

Experimental Work Hypotheses:

13. Samples Aligned with the Vertical Axis: Aligning samples with the vertical axis of the 3D printing machine results in consistent material properties and structural characteristics.
14. Average Values Considered: Utilizing average values from tested samples accurately represents the typical mechanical behavior of FDM 3D-printed components.
15. No Stress Concentration Caused by Machine Jaws: The experimental setup minimizes stress concentrations induced by machine jaws, providing accurate representations of material behavior.
16. Samples Tested at Room Temperature: Conducting tests at room temperature eliminates temperature-induced variations, offering a standardized assessment of material properties.
17. Constant Testing Speed: Maintaining a constant testing speed ensures consistent loading conditions, facilitating accurate comparisons of mechanical properties.
18. No Impact of 3D Printing Extrusion Process on Material Properties: The 3D printing extrusion process does not compromise the intrinsic material properties, allowing for the reliable evaluation of printed components.

By combining these hypotheses, we aim to thoroughly examine the relationship between FDM 3D printing, FEA simulations, and experimental observations, raising a comprehensive understanding of the structural characteristics of the investigated 3D-printed components.

## 3. Results and Discussion

### 3.1. Experimental Work

This work provides a detailed analysis of the impact of raster angles on structural integrity and failure behavior of 3D-printed material specimens. For this purpose, experimental and FEA simulations were used to examine the implications of raster angles on the mechanical performance and integrity of the material components. Three different raster angle samples, one set featuring intact specimens and the other consisting of samples with the introduced defects, were prepared to analyze the material samples at different raster angles. An open-source 3D printer was used to fabricate both sets of samples, utilizing biodegradable PLA material. The speed considered of 2 mm/min was used in the experimental setup. After preparing material samples, a universal testing machine was used for tensile testing of 3D-printed material samples.

**Figure 4:**
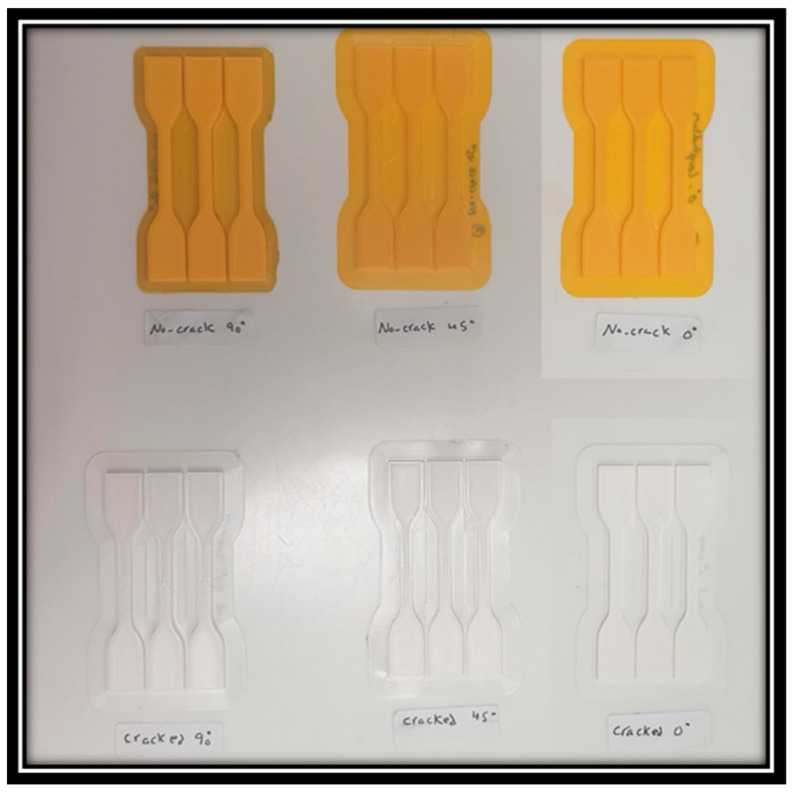
3D-printed material specimens.

Furthermore, this research delved into an extensive examination of various 3D-printed specimens, aiming to thoroughly assess their mechanical performance and potential failure mechanisms. Primarily, the specimens were subjected to tensile testing that involved applying tensile loads to the samples to analyze their capacity to endure and distribute stress along the axial direction. Figure 4 illustrates a 3D-printed specimen undergoing a tensile test.

The elastic modulus, a fundamental parameter in materials science and engineering, is a pivotal metric for assessing a material intrinsic stiffness or resistance to undergoing deformation when subjected to external forces. It is explicitly established that a material boasting a higher elastic modulus is inherently stiffer, signifying its greater capacity to maintain its shape and resist deformation when subjected to mechanical loads compared to a material with a lower elastic modulus. Equation (1) represents how the elastic modulus can be calculated:

(1) $$E\left[\mathrm{MPa}\right]=\frac{F\times L_{o}}{A\times \Delta L}$$

In the context of tensile testing, the elastic modulus (*E*) denotes the material capacity to deform under tension, measured in megapascals (MPa). The force (*F*) applied to the specimen during testing is expressed in newtons (N), while the original gage length (*L_o_*) refers to the initial length of the sample, measured in millimeters (mm). Additionally, the actual cross-sectional area (*A*) characterizes the sample material initial area in square millimeters (mm^2^), and the change in length (Δ*L*) represents the elongation in the sample length under longitudinal load, measured in millimeters.

**Figure 5:**
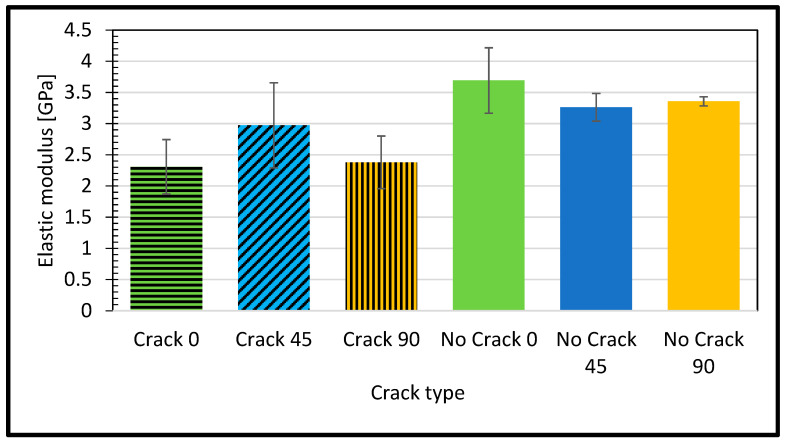
Elastic modulus for different raster angles for defective and intact samples.

Empirical analysis has revealed a noteworthy discrepancy in the elastic modulus between a sample devoid of cracks, oriented at a 45-degree angle, and a reference specimen. Specifically, the elastic modulus of the crack-free 45-degree microstructure measures at 3.26 GPa, a modest 0.3% higher than the established reference value of 3.25 GPa as shown in Figure 5.

**Figure 6:**
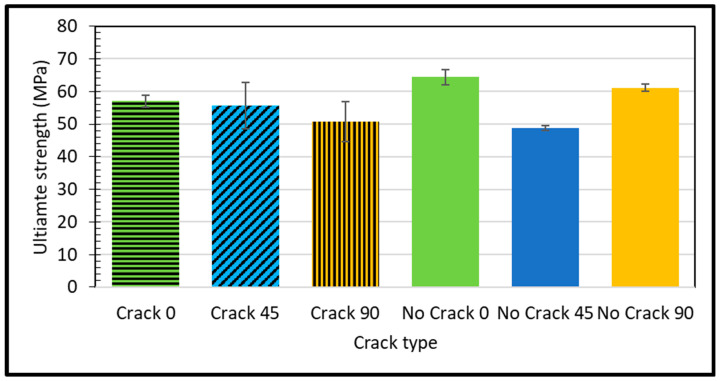
Ultimate strength for different raster angles for different samples.

The material with the pristine, crack-free 45-degree microstructure exhibits a slightly higher stiffness compared to the reference material. FDM is a popular technique for producing thermoplastic parts. Illustrated in Figure 6 is a graphical representation portraying the influence of specimens on the resistance to failure beyond the yield point. The determination of the ultimate tensile strength is conducted through the utilization of Equation (2):

(2) $$\mathrm{Ultimate}\;\mathrm{strength}\;\left(\mathrm{Pa}\right)=\frac{\;\mathrm{Maximum}\;\mathrm{load}\;\mathrm{measured}\;\left(N\right)}{\;\mathrm{Gauge}\;\mathrm{length}\;\mathrm{original}\;\mathrm{area}\;\left(m^{2}\right)}$$

Ultimate strength, a critical parameter in materials science, represents the maximum stress a material can withstand before failure. A higher ultimate strength indicates greater intrinsic fortitude. Empirical findings show a compelling divergence in ultimate strength between the pristine, crack-free 45-degree microstructure sample and a reference specimen. The former exhibits a 7% lower ultimate strength (48.82 MPa vs. 52.5 MPa), as shown in Figure 6.

The material with the unmarred 45-degree microstructure is weaker compared to the reference material, meaning it can break more easily under pressure. There are many reasons for this difference in strength, and more research is needed to understand them fully.

Defects within the material, such as structural irregularities, voids, or dislocations, can act as stress concentrators, leading to premature material failure. Elongation serves as a pivotal parameter in material science, signifying the ductile nature of a material, which is its capacity to undergo deformation without fracture. The computation of elongation is facilitated through the application of the subsequent Equation (3):

(3) $$\mathrm{Elongation}\;\left(\%\right)=\frac{L_{f}-L_{o}}{L_{o}}\times 100$$

where $L_{o}$ is the original gage length in mm, and $L_{f}$ is the final length at the break in mm.

**Figure 7:**
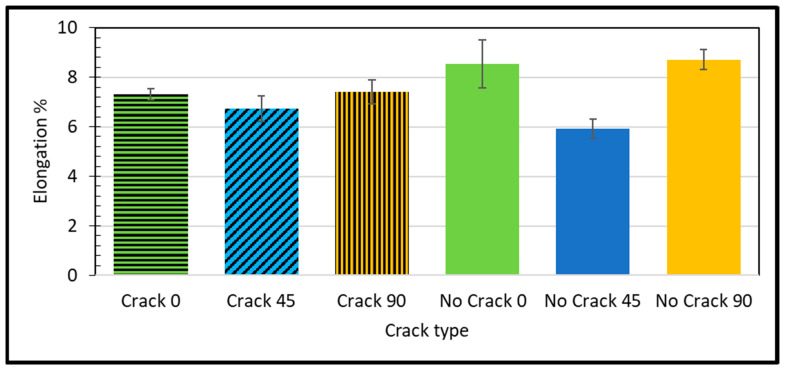
Estimated elongation % for different samples and raster angles. Observed defects in produced specimen.

Materials with higher elongation values are generally more ductile. Arifvianto et al. [41] studied the impact of extruder temperatures and raster orientations on the mechanical properties of 3D-printed PLA material. Elongation serves as a measure of a material resilience against mechanical deformation. Empirical evidence reveals a significant difference in elongation between the unblemished 45-degree microstructure sample and a reference specimen. The former exhibits an elongation of 5.93%, a notable 24% reduction compared to the reference value of 7.8%, as shown in Figure 7.

The significant decrease in elongation indicates that material with pristine 45-degree microstructure is less ductile compared to the reference material, making it more prone to fracture under deformation. The origins of this variance in elongation involve a complex interplay of factors that require further investigation. One possible explanation lies in the microstructural domain. The 45-degree microstructure might possess a internal arrangement, contributing to its reduced ductility. Afonso et al. [42] assessed the effect of 3D printing properties on the mechanical characteristics and mass of PLA objects and developed predictive models for the observed responses. Variations in orientation can significantly affect a material’s ability to deform without fracturing. Specific elements might make the material susceptible to brittle fracture under load. Finally, defects within material, such as voids lead to cracks, further contributing to the material’s reduced ductility.

The cracked sample at a 0-degree raster angle showed a significantly lower elastic modulus (2.308 GPa) compared to the no crack sample (3.69 GPa) at the same orientation. This indicates that the presence of a crack reduces the material’s stiffness. The ultimate strength of the cracked sample at a 0-degree raster angle was also lower (57.02 MPa) compared to the no crack sample (64.39 MPa). This suggests that the crack weakens the material’s overall strength. As highlighted by Janos Plocher et al. [43], the presence of a crack acts as a stress concentrator, amplifying stress levels around the crack area beyond what would typically occur in a crack-free material. This stress concentration contributes to the reduced stiffness and strength observed in the cracked sample. The increased stress concentration around the crack makes the material more susceptible to failure under loads that would not usually cause failure in a crack-free material. The difference in ultimate strength between the cracked and the no crack samples highlights the detrimental effect of a crack on a material’s strength. The elongation percentage of the cracked sample at a 0-degree raster orientation was lower (7.32%) compared to the intact sample (8.54%). This indicates that the presence of a crack reduces the material ductility, making it more prone to fracturing under deforming loads. As noted by Betül Gülçimen Çakan et al. [34], the raster angle influences a material’s tensile properties. A 0-degree raster angle provides the highest strength, while a 45-degree raster angle results in the most ductile behavior.

At a 45-degree raster orientation, the cracked sample showed a slightly lower elastic modulus (2.97 GPa) compared to the intact sample (3.26 GPa) at the same orientation. This indicates that the presence of a crack reduces the material’s stiffness. Interestingly, the ultimate strength of the cracked sample at a 45-degree raster orientation was higher (55.65 MPa) compared to the intact sample (48.82 MPa). This suggests that the crack, in this case, might have altered the material failure mechanism, leading to a higher ultimate strength [44]. However, it is important to note that cracks generally make materials more brittle, meaning they are less likely to deform before failure. The elongation percentage of the cracked sample at a 45-degree raster orientation was higher (6.73%) compared to the intact sample (5.93%). This suggests that the crack might have introduced some ductility to the material at this particular orientation.

On the other hand, the cracked sample at a 90-degree raster orientation showed a significantly lower elastic modulus (2.38 GPa) compared to the intact sample (3.36 GPa) at the same orientation. This indicates that the presence of a crack reduces the material’s stiffness. The ultimate strength of the cracked sample at a 90-degree raster orientation was also lower (50.75 MPa) than the intact sample (61.14 MPa). This suggests that the crack weakens the material’s overall strength. The elongation percentage of the cracked sample at a 90-degree raster orientation was lower (7.4%) than that of the intact sample (8.71%). This indicates that the presence of a crack reduces the material’s ductility, making it more prone to fracturing under deforming loads. As noted by Mohammed Algarni et al. [37], a 90-degree raster angle generally provides better strength and strain properties in PLA materials. However, the presence of a crack significantly diminishes these properties.

### 3.2. Mathematic Model of the Intact 3D-Printed Samples

A computational approach was presented to model damage in the context of the linear material behavior exhibited by 3D-printed components [35]. The approach involves extracting a unit cell from the microstructure of a single layer of the printed parts, commonly known as the representative volume element. Given that the layers of the printed parts exhibit orthotropic characteristics, the representative volume element is treated as an orthotropic material. Consequently, the stress–strain relationship governing an orthotropic material is demonstrated in this context:

(4) $$\left[\begin{array}{cccccc} C_{11} & C_{12} & C_{13} & 0 & 0 & 0\\ C_{12} & C_{22} & C_{23} & 0 & 0 & 0\\ C_{13} & C_{23} & C_{33} & 0 & 0 & 0\\ 0 & 0 & 0 & C_{44} & 0 & 0\\ 0 & 0 & 0 & 0 & C_{55} & 0\\ 0 & 0 & 0 & 0 & 0 & C_{66} \end{array}\right]\left\{\begin{array}{c} \varepsilon_{11}\\ \varepsilon_{22}\\ \varepsilon_{33}\\ \gamma_{23}\\ \gamma_{13}\\ \gamma_{12} \end{array}\right\};\;\mathrm{or}\;\left\{\sigma \right\}=\left[C\right]\left\{\varepsilon \right\}$$

The constitutive matrix, denoted as *C*, is integral to the analysis. The reciprocal of the mentioned equation yields the compliance matrix (*S*):

(5) $$\left\{\begin{array}{c} \varepsilon_{11}\\ \varepsilon_{22}\\ \varepsilon_{33}\\ \gamma_{23}\\ \gamma_{13}\\ \gamma_{12} \end{array}\right\}=\left[\begin{array}{cccccc} S_{11} & S_{12} & S_{13} & 0 & 0 & 0\\ S_{12} & S_{22} & S_{23} & 0 & 0 & 0\\ S_{13} & S_{23} & S_{33} & 0 & 0 & 0\\ 0 & 0 & 0 & S_{44} & 0 & 0\\ 0 & 0 & 0 & 0 & S_{55} & 0\\ 0 & 0 & 0 & 0 & 0 & S_{66} \end{array}\right]\left\{\begin{array}{c} \sigma_{11}\\ \sigma_{22}\\ \sigma_{33}\\ \tau_{23}\\ \tau_{13}\\ \tau_{12} \end{array}\right\};\;\mathrm{or}\;\left\{\varepsilon \right\}=\left[S\right]\left\{\sigma \right\}$$

The coefficients *S* matrix are as follows:

(6) $$S_{11}=\frac{1}{E_{1}},S_{12}=-\frac{v_{12}}{E_{1}},S_{13}=-\frac{v_{13}}{E_{1}},S_{22}=\frac{1}{E_{2}},S_{23}=-\frac{v_{23}}{E_{2}},\;S_{33}=\frac{1}{E_{3}},S_{44}=\frac{1}{G_{23}},S_{55}=\frac{1}{G_{13}},S_{66}=\frac{1}{G_{12}}$$

The representative volume element is assumed to be macroscopically homogeneous in the homogenization approach, and the mean stresses $\sigma_{ij}$ and mean strains $\varepsilon_{ij}$ are estimated using the following equations:

(7) $$\sigma_{ij}=\frac{1}{V_{\mathrm{RVE}}}\int_{V}\sigma_{ij}\left(x_{1},x_{2},x_{3}\right)dV,\varepsilon_{ij}=\frac{1}{V_{\mathrm{RVE}}}\int_{V}\varepsilon_{ij}\left(x_{1},x_{2},x_{3}\right)dV$$

The mean stress and strain domains are expressed as follows:

(8) $$\varepsilon_{ij}=v_{f}\varepsilon_{ij}^{f}+v_{m}\varepsilon_{ij}^{m}$$

(9) $$\sigma =v_{f}\sigma_{ij}^{f}+v_{m}\sigma_{ij}^{m}$$

In the description context, f represents the fiber, and m signifies the matrix material. The elastic constitutive relation for the homogenized representative volume element is expressed as follows:

(10) {σ}=[C]{ε}

The determination of the constitutive matrix *C*, as outlined in Equation (10), is carried out through a methodology detailed in [36]. Finite element models for the representative volume element are formulated to implement a numerical homogenization approach for deriving the coefficients of the effective stiffness matrix *C*, specifically for the layers constituting 3D-printed components. Additional information regarding the computational models applied in the context of linear material modeling can be found in [36].

### 3.3. Mathematical Model of the Defective 3D-Printed Samples

In this analysis, finite element analysis was conducted adopting ANSYS package to investigate the behavior of the intact and the defective samples under tensile conditions, imposing the loads and the constraints analogously to the experimental setup. It has been addressed that the layer-wise approach employed in the FDM printing process fundamentally results in non-isotropic characteristics in the produced parts. Consequently, the 3D-printed specimens have been represented in the model as a homogenous and orthotropic material. This representation involves employing a tailored set of engineering constants corresponding to each printing parameter, as derived from the outcomes of the conducted tensile tests. The expression of the constitutive law is manifested through the stiffness matrix outlined in Equation (11), adhering to Hooke’s law [37]. In this equation, *σ* and *ε* represent the stress and strain vectors, respectively, while *D* signifies the stiffness tensor:

(11) $$\left\{\begin{array}{c} \sigma_{x}\\ \sigma_{y}\\ \sigma_{z}\\ \tau_{xy}\\ \tau_{yz}\\ \tau_{xz} \end{array}\right\}=\left[\begin{array}{cccccc} D_{11} & D_{12} & D_{13} & 0 & 0 & 0\\ D_{21} & D_{22} & D_{23} & 0 & 0 & 0\\ D_{31} & D_{32} & D_{33} & 0 & 0 & 0\\ 0 & 0 & 0 & D_{44} & 0 & 0\\ 0 & 0 & 0 & 0 & D_{55} & 0\\ 0 & 0 & 0 & 0 & 0 & D_{66} \end{array}\right]\cdot \left\{\begin{array}{c} \varepsilon_{x}\\ \varepsilon_{y}\\ \varepsilon_{z}\\ \gamma_{xy}\\ \gamma_{yz}\\ \gamma_{xz} \end{array}\right\}$$

In general, the mathematical model assumes a uniform material composition, and every layer, including samples, is considered a homogeneous material featuring 100% infill. The material properties were averaged across regions with both infill and voids. A mesh and elements analogous to those used for intact samples were employed for defective samples. The 3D-printed samples exhibit localized weak points aligned with inter-layer surfaces, where perfect adhesion is assumed. Alternatively, a cohesive model, characterized by elastic properties defined by the constitutive law in Equation (12) [38], can be applied to cohesive surface elements. Here, *σ* denotes the stress vector specific to the local context, *ε* represents the vector compiling the separations within the cohesive element, *K* stands for the stiffness matrix, and *nst* corresponds to the local coordinate system:

(12) $$\left\{\begin{array}{c} \sigma_{n}\\ \sigma_{s}\\ \sigma_{t} \end{array}\right\}=\left[\begin{array}{ccc} K_{nn} & 0 & 0\\ 0 & K_{ss} & 0\\ 0 & 0 & K_{tt} \end{array}\right]\cdot \left\{\begin{array}{c} \varepsilon_{n}\\ \varepsilon_{s}\\ \varepsilon_{t} \end{array}\right\}$$

The cohesive element is obviously recognized as the surface between layers perpendicular to the applied load. The stiffness factors, namely *K_nn_*, *K_ss_*, and *K_tt_*, align with the diagonal elements of the material stiffness matrix as articulated in Equation (11). This alignment results from the cohesive model representing the bonding within the base material, where the Tsai-Wu model can be adopted to recognize the starting point of the fracture initiation [39]. This method proves to be a suitable strategy for simulating 3D-printed components due to their inherent non-isotropic characteristics arising from the layer-wise manufacturing process. Considering 3D-printed components produced through FDM as laminate composite structures renders traditional failure models, like Tresca, unsuitable. The application of the Tsai-Wu model involves conceptualizing each layer formed in the 3D printing process as a thin plate experiencing plane stress [41,42,43], utilizing the classical orthotropic laminate model. The fundamental assumption entails the presence of a failure surface defined by Equation (13), where the factors *F_ij_* are defined as per Equation (14) [39]:

(13) $$F_{1}\sigma_{11}+F_{2}\sigma_{22}+F_{11}\sigma_{11}^{2}+F_{22}\sigma_{22}^{2}+F_{66}\sigma_{12}^{2}+2F_{12}\sigma_{11}\sigma_{22}=1$$

(14) $$F_{1}=\frac{1}{f_{11}^{T}}-\frac{1}{f_{11}^{C}}F_{11}=\frac{1}{f_{11}^{T}\cdot f_{11}^{C}}F_{12}=-\frac{1}{2}\sqrt{F_{11}\cdot F_{22}}\;F_{2}=\frac{1}{f_{22}^{T}}-\frac{1}{f_{22}^{C}}F_{22}=\frac{1}{f_{22}^{T}\cdot f_{22}^{C}}F_{66}=\frac{1}{f_{12}^{2}}$$

In the aforementioned equation, $f_{ii}$ is the strength of the material studied considered along the *i* direction throughout tension ($f_{u}^{T}$) and compression ($f_{u}^{C}$) examinations, and $f_{ij}$ is the pure shear strength alongside the *ij* plane. Through an experiment, $f_{11}^{T}$, $f_{11}^{C}$, $f_{22}^{T}$, $f_{22}^{C}$ that could be evaluated via uniaxial tensile tests with 3D-printed tensile test samples with 0° and 90° orientation. The $f_{12}$ value can be estimated from the failure strength value $f_{\theta }^{T}$ for the investigated printed angle of the sample. By applying the tensor transformation law described in Equation (15) [39], $f_{12}$ can be calculated by substituting Equation (15) into Equation (14):

(15) $$\begin{array}{l} \sigma_{11}=f_{\theta }^{T}\cos^{2}\theta \\ \sigma_{22}=f_{\theta }^{T}\sin^{2}\theta \\ \sigma_{12}=-f_{\theta }^{T}\sin\theta \cos\theta \end{array}$$

### 3.4. Finite Element Analysis (FEA)

**Figure 8:**
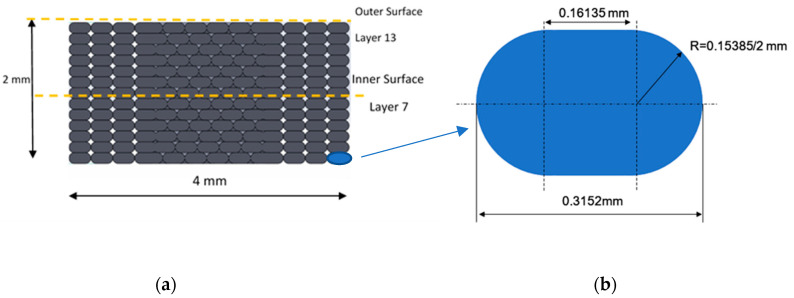
(**a**) Modeling dimensions considered in the FE for the 3D-printed filament; (**b**) section details and configuration used in the FEA.

Finite element analysis (FEA) of 3D-printed material specimens was performed to examine the mechanical behavior, structural integrity, and failure of material specimens prepared at different raster angles. The first stage is to generate a 3D CAD model of all models with various raster angles that will be evaluated. Figure 8 shows the cross-sectional shape of the deposited filaments, which is close to an elliptic curve.

**Figure 9:**
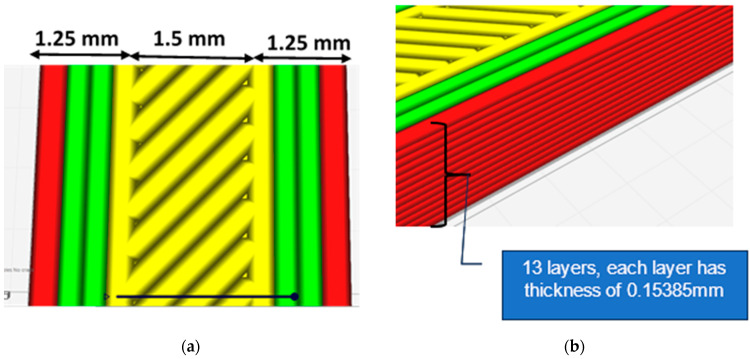
(**a**) Dimensions considered for the specimen modeling; (**b**) slicing of thickness of the sample.

The detailed dimensions parameters for the specimen are illustrated in Figure 9. Subsequently, specimens were extruded layer by layer to achieve a number of layers of 13, regarding the thickness and width of each layer as shown.

**Figure 10:**
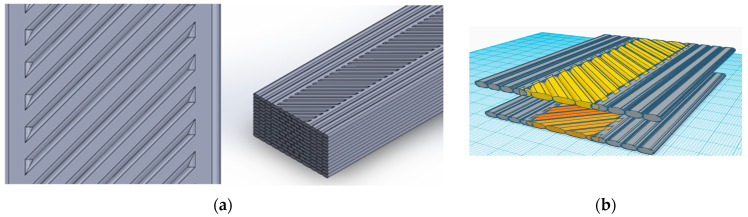
(**a**) Modeling the FE overlap; (**b**) sample of the sequence of the 3D modeling process.

The models have 13 layers with a thickness of 0.15385 mm each, a group of strings that overlap by 0.001 mm to ensure no gaps, and a layer sequence. Figure 10 illustrates the molded material layer by layer on the bed and the scheme of different orientation types.

**Figure 11:**
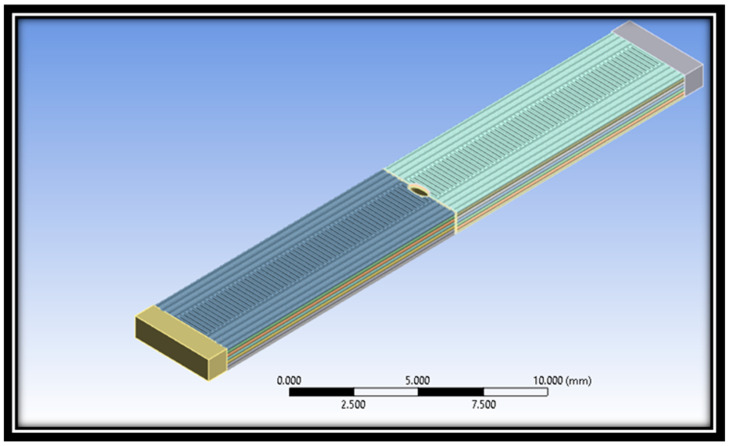
CAD model of material specimen at raster angle 90°.

The 3D CAD model of the 3D-printed crack material sample at a raster angle 90° is shown in Figure 11. In order to get the preferred outcomes, making a CAD model with proper dimensions is essential.

**Figure 12:**
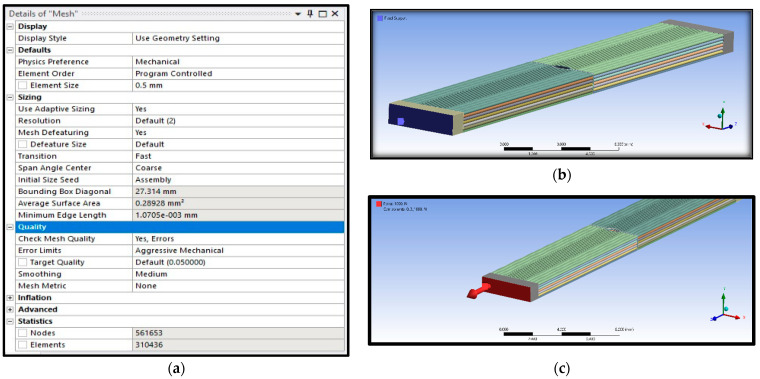
(**a**) Mesh details, (**b**) fixed support, and (**c**) force applied.

In addition, meshing is also an essential parameter to get accurate and precise findings. Meshing is the primary step in FEA simulation after the creation of geometry. In this FEA simulation, a tetrahedral mesh was used due to some complexity in the material samples. The boundary conditions with mesh details are shown in Figure 12.

**Figure 13:**
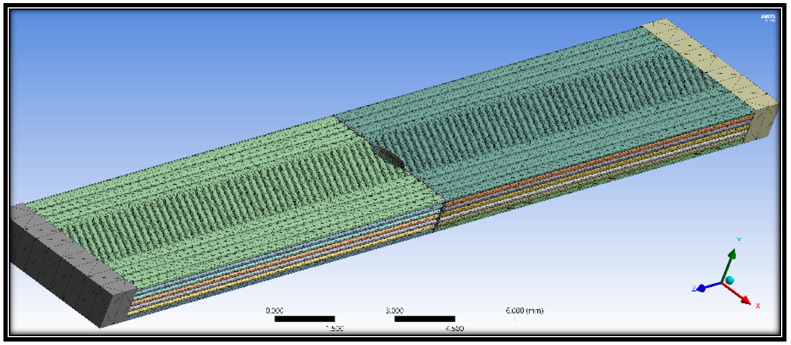
Patch conforming mesh method applied.

The applied mesh method can be seen below in Figure 13.

The applied load of 1 KN was used in FEA simulation and fixed on the other side. Table 3 lists the mesh statistics of material samples at different raster angles for intact and defective cases. It can be observed from the mesh statistics that the number of elements decreases with the increase in raster angles. The highest number of elements and nodes were observed for raster angle 0°, and the lowest number of elements and nodes were examined for raster angle 90°.

**Figure 14:**
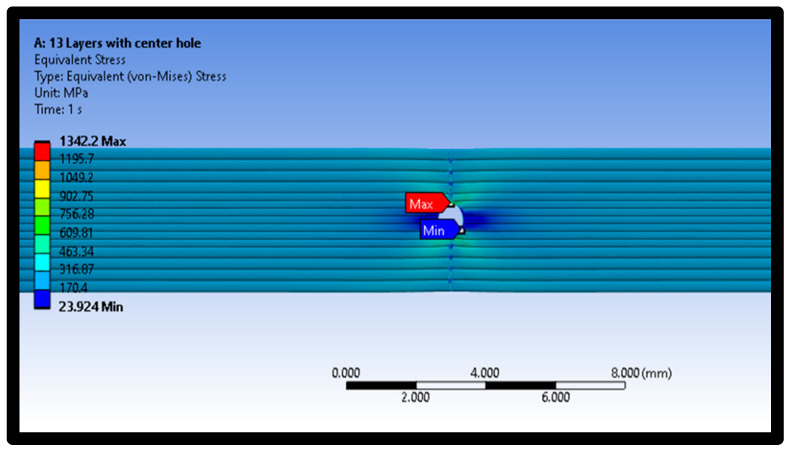
Distribution of Von Mises stress in defective sample at raster angle of 90°.

**Figure 15:**
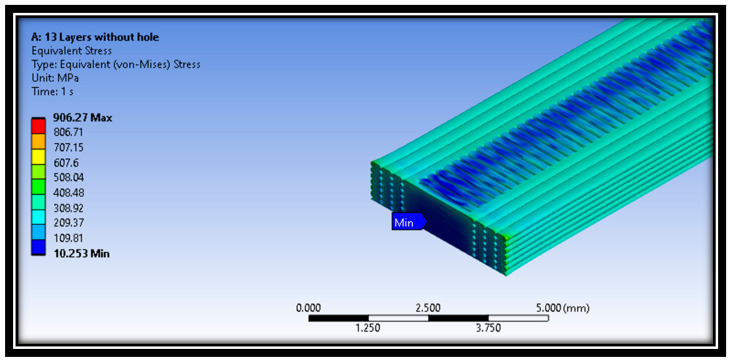
Distribution of Von Mises stress in an intact sample at raster angle 90°.

**Figure 16:**
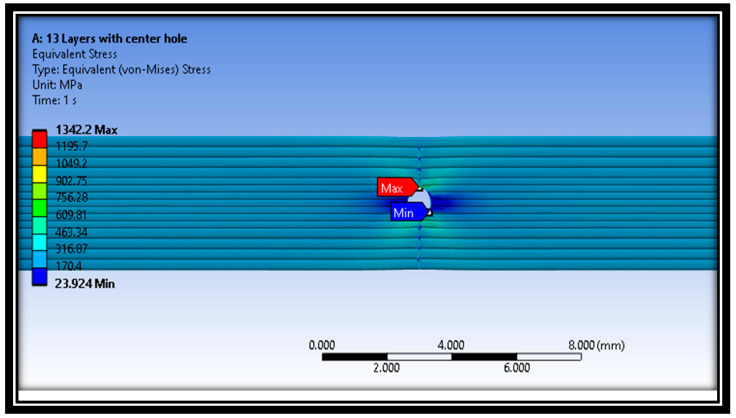
Distribution of Von Mises stress for defective sample at raster angle 0°.

Moreover, Figure 14, Figure 15 and Figure 16 represent the distribution of Von Mises stresses in intact and defective material samples at raster angles of 0° and 90°. It is evident from Figure 14 that maximum stress concentration was seen at the edges of the central hole, while minimum stress was observed at the middle layer of the material samples. 

In addition, the distribution of Von Mises stresses for the intact sample at a raster angle of 90° for layer 13 is also shown in Figure 14. The minimum stress was examined at the middle layer 13 of the material sample. The maximum and minimum Von Mises stresses values are 906.27 MPa and 10.253 MPa, respectively. On the other hand, the distribution of Von Mises stresses for defective samples at a raster angle of 0° is also shown in Figure 16. The results show that maximum and minimum stress values are at the material samples’ central portion. 

**Figure 17:**
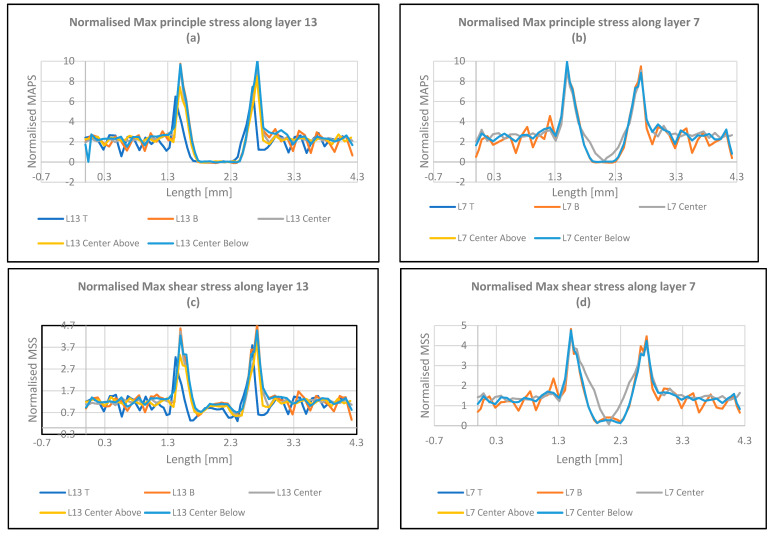

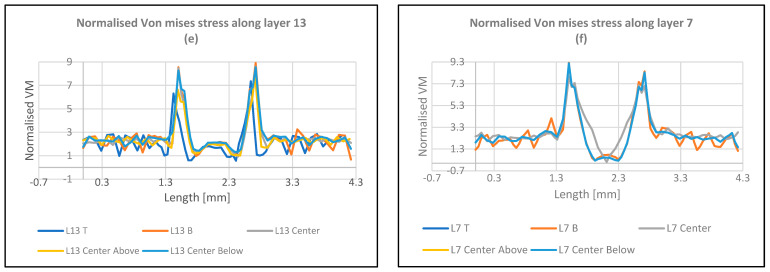
Graphical representations of normalized Max principal, Max Shear, and Von Mises stresses along layers 13 and 7 for the defective samples at a raster angle 45°.

Figure 17 illustrate the distribution of principal, shear, and Von Mises stresses across various layers in defective samples with a 45° raster angle. The maximum principal stress peaks at x = 1.5, coinciding with the void termination and the stress concentration region. Additionally, the highest stress levels are observed at x = 1.5 and x = 2.75 upon applying an external force, suggesting that crack propagation initiates at the void edges. Comparing the maximum principal and shear stresses within intact material at x = 1.5 reveals that the maximum principal stress is twice the magnitude of the maximum shear stress. This observation is crucial as it occurs at the void edge and exceeds the Von Mises stress level. Moreover, layers 7 and 13 exhibit identical stress values due to uniform material stretching, indicating uniform stress distribution within these layers. The difference between maximum principal and shear stresses in this location underscores its importance. The maximum principal stress is the dominant stress component at the void periphery and is most likely to initiate cracks. Comparing stress distribution between 45-degree and 90-degree raster patterns reveals that the 45-degree pattern exhibits lower principal stress values. This suggests that the 45-degree pattern is structurally more robust and less susceptible to failure than the 90-degree pattern due to its more uniform stress distribution. The 45-degree pattern’s superior structural integrity stems from its more even stress distribution, making it less prone to failure than the 90-degree pattern. The maximum shear and Von Mises stresses peak at x = 1.5, coinciding with the void termination point, indicating the critical nature of this region where stress concentrations are most prominent. Additionally, under applied force, the highest stress levels occur at x = 1.5 and x = 2.75, suggesting that crack propagation initiates at the void extremities. Comparing maximum shear stress with Von Mises stress within intact material at x = 1.5 reveals that the maximum shear stress is approximately half the magnitude of the Von Mises stress. This observation is crucial as it occurs at the void edge and is lower than the maximum principal stress. Comparing stress distribution graphs for 45-degree and 90-degree raster patterns shows that the 45-degree pattern exhibits lower shear stress and Von Mises stress values. This suggests that the 45-degree pattern is structurally more robust and less prone to failure than the 90-degree pattern due to its more uniform stress distribution [17].

Eventually, in this investigation, experiments and FEA were conducted to study the impact of the raster angle on the integrity of the defective 3D-printed parts. The summary of the study is shown in the tables below.

For the experimental work, as illustrated in Table 4, the following are observed:

- The defective sample stiffness (modulus of elasticity) demonstrated lower levels than the corresponding intact ones. The lowest stiffness is at the raster angle of 90 degrees, and the highest value is at 0 degrees, the longitudinal raster. This is because the longitudinal raster will support the sample resistance to the load contrary to the transverse one.
- A similar conclusion to the previous point has been reached regarding the ultimate tensile strength of the defective sample, where all samples showed less resistance to failure than the intact ones. Furthermore, the highest tensile strength is indicated by the longitudinal raster. The lowest resistance to the failure is at the transverse raster, i.e., 90 degrees.
- The elongation for the defective samples is lower than the intact ones, which is normal. In addition, it has been observed that the longitudinal raster offers the lowest elongation due to having the highest stiffness in this direction, whereas the transverse raster accommodates the most elevated extension due to the insufficient stiffness.

Regarding the FEA results as depicted in Table 5, we can conclude the following observations:

- The stresses at the outer layer (layer 13), demonstrated higher levels than the inner layer, i.e., layer 7, since the outer layer is not supported by one side, whereas the inner layer is supported by material from both sides.
- The maximum principal stresses, max shear stresses, and Von Mises stresses for the defective samples are higher than the analogous values for the intact samples. This indicates the complication of the case and that the raster angle plays a significant role in the failure’s contribution.
- The sample always shows the highest stress at the longitudinal raster, and this will lead to quick failure.

## 4. Conclusions

Our study investigates the impact of raster angles on the structural integrity and failure behavior of 3D-printed material specimens. The research combines experimental methods using the Fused Deposition Method (FDM) with numerical simulations employing the linear elastic finite element method. Two sample groups are utilized, one with flawless specimens and the other with introduced defects. The finite element analysis (FEA) employs highly detailed models to accurately represent the geometry and dimensions of the 3D-printed filament. The study conducts a comprehensive comparative analysis between experimental findings and FEA simulations, leading to detailed conclusions:

1. As demonstrated graphically, stress distribution remains consistent throughout inner layers in scenarios with uniform layers and no voids. The absence of voids typically leads to initial fractures occurring at the terminal regions of the cross-sectional profile.
2. In situations devoid of voids, maximum principal stresses surpass both shear and Von Mises stresses. Specifically, the 45-degree raster pattern exhibits the highest stress levels compared to the 0- and 90-degree raster patterns in void-free cases.
3. Stress concentration in cases involving voids primarily occurs at locations x = 1.5 and 2.75, corresponding to the terminus of the void.
4. In configurations with voids, the 90-degree raster pattern demonstrates the highest stress values compared to the 0- and 45-degree raster patterns.
5. Maximum principal stresses hold a significant dominance over both shear and Von Mises stresses in samples without voids. This emphasizes their primary role in shaping stress distributions in void-free configurations.
6. For void-free cases, stress magnitudes vary based on the orientation of the load, particularly notable with the 45-degree raster pattern. Compared to the 0- and 90-degree raster configurations, the utilization of 45-degree raster results in the highest stress magnitudes, highlighting the significant influence of load orientation on stress patterns.
7. Stress concentrations reach their peak at specific spatial coordinates, notably at x = 1.5 and 2.75, corresponding to the extremities of the voids. These locations emerge as focal points of heightened stress concentration within configurations that include voids.
8. Cracks typically initiate precisely at the extremities of the cross-section. This observation suggests a trend toward end-point fracture initiation in configurations devoid of voids. This is when with no voids exist within the cross-sectional geometry.
9. The analysis thoroughly examines stress distribution and fracture behavior across various structural configurations. It demonstrates how stress patterns vary depending on void presence and load orientation. In void-free scenarios, maximum principal stresses are pivotal in determining structural integrity. The identification of stress concentration zones, particularly at void termini, offers valuable insights into potential failure points. Additionally, the observation that cracking initiates at cross-sectional ends highlights a significant trend in fracture behavior, suggesting localized stress accumulation. These findings underscore the importance of considering structural characteristics and load orientations in engineering analysis and design processes. Understanding stress distributions and fracture mechanisms enables engineers to make informed decisions, enhancing material and structural performance and durability.
10. In the experimental tensile test, we observed that the highest values for elastic modulus, ultimate strength, and elongation were recorded for the “No Crack 0” configuration. Conversely, the lowest values for elastic modulus, ultimate strength, and elongation were observed for the “No Crack 0”, “Crack 90”, and “No Crack 45” configurations, respectively.

## Figures and Tables

**Table 1:** Material properties of PLA [39].

Considered Properties	Experimental Characteristics
Tensile Modulus	2.35 GPa
Yield Strength	50 MPa
Strength at Break	46 MPa
Elongation at Break	5.3%

**Table 2:** Standard Ultimaker S5 printing parameter used.

Parameters Used	Settings
Printing speed	1.167 mm/s
Temperature of the printing	205 °C
Temperature of the printing bed	65 °C
Height of the printed layer	0.2 mm
Thickness of the wall	1 mm
Thickness of the top layer	1 mm
Thickness of the bottom layer	1 mm

**Table 3:** Mesh statistics for FEA analysis.

Scheme 0.	Abbreviation	Number of Elements	Number of Nodes
Intact raster angel 0	Intact R0	350,908	618,796
Intact raster angel 45	Intact R45	348,205	611,695
Intact raster angel 90	Intact R90	256,805	452,647
Crack raster angel 0	Crack R0	360,480	641,234
Crack raster angel 45	Crack R45	354,160	630,487
Crack raster angel 90	Crack R90	266,220	480,370

**Table 4:** Summary of the experimental work.

Experimental	Modulus of Elasticity (E)	Tensile Strength (TS)	Elongation (EL%)
Intact R0	1.14	1.23	0.76
Intact R45	1.03	1.16	1.09
Intact R90	1.00	1.06	1.12
Crack R0	0.90	1.09	0.87
Crack R45	0.73	0.97	0.94
Crack R90	0.71	0.93	0.95

**Table 5:** Results summary between the inner and outer surfaces.

	Inner Surface (Layer 7)			Outer Surface (Layer 13)		
FEA	Max Principal	Max Shear	Von Misses	Max Principal	Max Shear	Von Misses
Intact R0	3.9	1.3	2.3	5	1.8	3.9
Intact R45	4.3	1.34	2.37	5.8	2.1	3.95
Intact R90	4.8	1.4	2.5	6.5	2.3	4
Crack R0	9.8	4.7	8.3	9.4	4.3	8.2
Crack R45	9.9	4.8	9.2	10.1	4.8	8.9
Crack R90	12.6	6.0	10.6	12.1	5.7	10.4

## Data Availability

Data are contained within the article.
